# Supplementary material for: Association between Outdoor and Indoor Air Pollution Sources and Atopic Eczema among Preschool Children in South Africa
Source: Int J Environ Res Public Health. 2024 Mar 11;21(3):326. doi: 10.3390/ijerph21030326 (PMC10969843; doi:10.3390/ijerph21030326)
Supplement: Supplementary file 1 [file ijerph-21-00326-s001.zip › ijerph-2848449-supplementary.pdf]

Supplementary materials

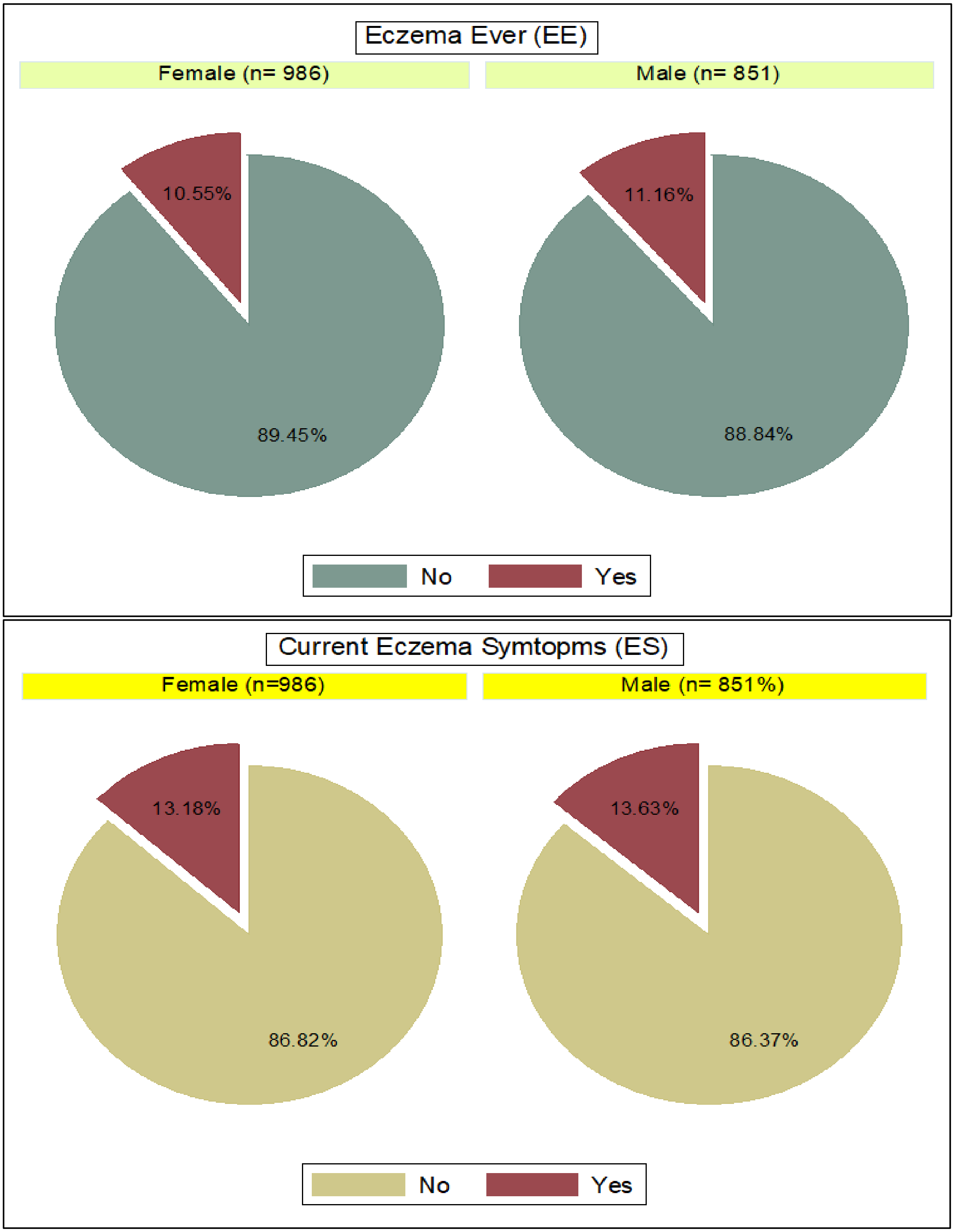

Figure S1: Prevalence of eczema ever (EE) and current eczema symptoms (ES) by children's sex (male and female), (n=1840).

Table S1: Overall prevalence of eczema ever (EE) and current eczema symptoms (ES), (n=1840).

	Total	Percentage
Ever had eczema (EE)		
Yes	199	11.9
No	1475	88.1
Current eczema symptoms ES		
Yes	246	13.37
No	1594	86.63

Table S2: The prevalence of having current ES among the participants, along with crude and adjusted ORs.

Variable	Total* (%)	Crude OR (95% CI) †	P-value	Adjusted OR (95% CI) †	P-value
**Sex**					
Female	130 (52.8)	1		1	
Male	116 (47.2)	1.04 (0.79–1.36)	0.779	0.99 (0.75–1.32)	0.962
**Hours spent watching television per week**					
Less than 1 hour	54 (22.0)	1		1	
1 hour to less than 3 hours	95 (38.6)	0.90 (0.62-1.29)	0.564	0.96 (0.65–1.42)	0.848
3 hours or more	95 (38.6)	0.62 (0.43–0.89)	**0.009**	0.64 (0.43; 0.95)	**0.029**
**Residential cooking/heating fuel type**					
Electricity	199 (80.9)	1		1	
Gas	25 (10.2)	1.99 (1.25-3.18)	**0.004**	1.63 (1.00-2.65)	**0.052**
Open fire/Paraffin	19 (7.7)	3.25 (1.85–5.70)	**<0.001**	1.94 (1.00–3.74)	**0.049**
**Smoking exposure at home in the past 30 days**					
No	163 (66.3)	1		1	
Yes	60 (24.4)	1.87 (1.35-2.59)	**<0.001**	1.61 (1.07; 2.43)	**0.022**
**Smoking exposure at school in the past 30 days**					
No	181 (73.6)	1		1	
Yes	5 (2.0)	1.84 (0.68–4.97)	0.227	0.81 (0.27–2.39)	0.701
**Mother/female guardian smoke cigarette**					
no	226 (91.9)	1		1	
yes	20 (8.1)	1.44 (0.87–2.38)	0.156	1.23 (0.71–2.13)	0.451
**Father/male guardian smoke cigarette**					
no	174 (70.7)	1		1	
yes	71 (28.9)	1.20 (0.89–1.61)	0.242	0.96 (0.67–1.38)	0.816
**Type of house**					
brick	196 (79.7)	1		1	
combination	12 (4.9)	1.12 (0.60–2.10)	0.720	1.07 (0.56-2.05)	0.838
corrugated iron	27 (11.0)	2.18 (1.38–3.44)	**0.001**	1.64 (0.99- 2.73)	**0.054**
mud	1 (0.4)	0.78 (0.10-6.18)	0.813	0.78 (0.09–6.63)	0.820
other	7 (2.8)	1.89 (0.81–4.41)	0.142	1.54 (0.60-3.98)	0.367
**Mode of transport to school**					

combination	13 (5.3)	1		1	
motor car	50 (20.3)	2.50 (1.33-4.71)	**0.005**	2.27 (1.19-4.34)	**0.013**
taxi/bus	55 (22.4)	3.15 (1.68–5.92)	**<0.001**	3.11 (1.63–5.95)	**0.001**
walk	119 (48.4)	2.82 (1.56–5.10)	**0.001**	2.68 (1.46-4.95)	**0.002**
other	9 (3.7)	6.01 (2.33–15.47)	<0.001	4.57 (1.67–12.51)	0.003
**Frequency of trucks passing near residence on weekdays**					
Never	59 (24.0)	1		1	
Almost all day	34 (13.8)	2.53 (1.56–4.09)	**<0.001**	2.08 (1.24-3.49)	**0.005**
Frequently through the day	32 (13.0)	0.57 (0.36–0.90)	**0.016**	0.52 (0.32-0.84)	**0.007**
Seldom	115 (46.7)	1.09 (0.78–1.53)	0.603	1.15 (0.81; 1.64)	0.429

*Total for each risk factor is different due to differences in missing values.
†Model adjusted for all the variables.
Values that are statistically significant at less than 0.02 for the crude OR and less than 0.05 for the adjusted OR are in bold font.
Current eczema symptoms (current ES).

Instructions for completing the questionnaire.

Examples of instructions for completing the questionnaire and demographic questions are given below.

On this sheet are questions about your child's name, school, and birth dates. Please write your answers to these questions in the space provided.

All other questions require you to tick your answer in a box. If you make a mistake put a cross in the box and tick the correct answer. Tick only one option unless otherwise instructed.

Examples of how to mark questionnaires: Age [4] years

To answer Yes/No, put a cross in the appropriate box as per the example below:

YES	
NO	**X**

SUBURB/ TOWNSHIP/SECTION WHERE YOUR CHILD LIVES: [ ]

YOUR CHILD'S SCHOOL: [ ]

TODAY'S DATE: [ ]

YOUR CHILD'S NAME: [ ]

YOUR CHILD'S AGE: [ ] years/month

YOUR CHILD'S DATE OF BIRTH: ☐

WEIGHT OF YOUR CHILD? ☐ kg

HEIGHT OF YOUR CHILD? ☐ Metres /centimeters (Please circle the measurement you used)

(Tick all your answers for the rest of the questionnaire)

Is your child a: ☐ **MALE** ☐ **FEMALE**

Core questionnaire for asthma

1. Has your child <u>ever</u> had wheezing or whistling in the chest at any time in the past?

YES	
NO	

IF YOU HAVE ANSWERED "NO" PLEASE SKIP TO QUESTION 6

2. Has your child had wheezing or whistling in the chest <u>in the past 12 months?</u>

YES	
NO	

IF YOU HAVE ANSWERED "NO" PLEASE SKIP TO QUESTION 6

3. How many attacks of wheezing has your child had in the <u>past 12 months?</u>

NONE	
1 TO 3	
4-14	
More than 12	

4. <u>In the past 12 months</u>, how often, on average, has your child's sleep been disturbed due to wheezing?

Never woken with wheezing	
Less than one night per week	
One or more nights per week	

5. In the past 12 months, has wheezing ever been severe enough to limit your child's speech to only one or two words at a time between breaths?

YES	
NO	

→ 6. Has your child <u>ever</u> had asthma?

YES	
NO	

7. Was the asthma diagnosed by a medical doctor or nurse?

YES	
NO	

8. In the past 12 months, has your child's chest <u>ever</u> sounded wheezy during or after playing?

YES	
NO	

9. In the past 12 months, has your child had a dry cough at night, apart from a cough associated with a cold or chest infection?

YES	
NO	

Core questionnaire for rhinitis

10. Has your child <u>ever</u> had a problem with sneezing, or a runny, or blocked nose when she/he DID NOT have a cold or the flu?

YES	
NO	

IF YOU HAVE ANSWERED "NO" PLEASE SKIP TO QUESTION 15

11. In the <u>past 12 months</u>, has your child had a problem with sneezing, or a runny, or blocked nose when she/he DID NOT have a cold or the flu?

YES	
NO	

IF YOU HAVE ANSWERED "NO" PLEASE SKIP TO QUESTION 15

12. In the <u>past 12 months</u>, has this nose problem been accompanied by itchy-watery eyes?

YES	
NO	

13. In which of the <u>past 12 months</u> did this nose problem occur? (Please tick any which apply)

January	
February	
March	
April	
May	
June	

July	
August	
September	
October	
November	
December	

14. In the past 12 months, how much did this nose problem interfere with your child's daily activities?

Not at all	
A little	
A moderate amount	
A lot	

→ 15. Has your child ever had hayfever?

YES	
NO	

16. Was the hayfever diagnosed by a doctor or nurse?

YES	
NO	

Core questionnaire for eczema

17. Has your child ever had an itchy rash, which was coming and going for at least six months?

YES	
NO	

IF YOU HAVE ANSWERED "NO" PLEASE SKIP TO QUESTION 23

18. Has your child had this itchy rash at any time in the past 12 months?

YES	
NO	

IF YOU HAVE ANSWERED "NO" PLEASE SKIP TO QUESTION 23

19. Has this itchy rash at any time affected your child on any of the following places:

the folds of the elbows, behind the knees, in front of the ankles, under the buttocks, or around the neck, ears or eyes?

YES	
NO	

20. At what age of the child did this itchy rash first occur?

Age 2-4 years	
Age 5 or more	
Can't remember	

21. Has this rash cleared completely at any time during the past 12 months?

YES	
NO	

22. In the past 12 months, how often, on average, has your child been kept awake at night by this itchy rash?

Never in the past 12 months	
Less than one night per week	
One or more nights per week	

→ 23. Has your child ever had eczema?

YES	
NO	

24. Was the eczema diagnosed by a doctor or nurse?

YES	
NO	

General questionnaire

1. How long has your child lived in this SUBURB/ TOWNSHIP/SECTION?

Less than 6 months	
6 to 12 months	
1 to 2 years	
3 years or longer	

2. How does your child usually get to school?

Walk	
Taxi/Bus	
Motor car	
Combination	
Other	

3. How far is the nearest Clinic or Hospital from your home?

15 minutes' walk or 5 minute drive	
1 hour walk or 15 minute drive	
more than an hour's walk or more than 30 minute drive	

4. What type of house does your child live in?

Brick	
Mud	
Corrugated iron	
Combination	
Other	

5. How many rooms are in your house? (Excluding bathrooms)

6. Do you have running water in the house?

YES	
NO	

7. In the past 12 months, how often, on average, did your child eat or drink the following? (Please leave blank if you do not know what a food is)

**Type of food**	**Never or occasionally**	**Once or twice per week**	**Three or more times per week**
Meat (e.g. beef, lamb, chicken, pork)			
Seafood (including fish)			
Fruit			
Vegetables (green and root)			
Pulses (peas, beans, lentils)			
Cereal (including bread)			
Pasta			
Rice			
Butter			
Margarine			
Nuts			
Potatoes			
Milk			
Eggs			
Fast food/burgers			

8. In the past 6 months, how often has your child been absent from school?

Never or occasionally	
Once or twice per week	
Three or more times a week	

9. During a normal week, how many hours a <u>day (24hours)</u> does your child watch television?

Less than 1 hour	
1 hour but less than 3 hours	
3 hours but less than 5 hours	
5 hours or more	

10. In your house, what fuel is usually used for cooking?

Electricity	
Gas	
Paraffin	
Open fires	
Other – Please specify	

11. In your house, what fuel is usually used for heating?

Electricity	
Gas	
Paraffin	
Open fires (wood,coal)	
Other – Please specify	

12. In the past 12 months, how often, on average, have you given your child paracetamol (e.g. Panadol, Pamol)?

Never	
At least once a year	
At least once per month	

13. How many older brothers and sisters does your child have?

Brothers	
Sisters	

14. How many younger brothers and brothers sisters does your child have?

Brothers	
Sisters	

15. Was your child born in this township/suburb?

YES	
NO	

16. How often do trucks pass through the street where you live, on weekdays?

Never	
Seldom	
Frequently through the day	
Almost all day	

17. Do you currently have a cat in your home?

YES	
NO	

18. In the past 12 months, have you had a cat in your home?

YES	
NO	

19. Do you currently have a dog in your home?

YES	
NO	

20. In the past 12 months, have you had a dog in your home?

YES	
NO	

21. Does the child's mother (or female guardian) smoke cigarettes?

YES	
NO	

22. Does the child's father (or male guardian) smoke cigarettes?

YES	
NO	

23. How many people living in the same house as your child smoke cigarettes?

[ ] People

24. In the past 30 days about how many days would you say your child was in a place where someone smoked close to him/her (no complete physical barrier i.e. smoke got to him/her)?

	Never	1-6 days	7-10 days	16-20 days	More than 20 days
At home					
At school					
In the car or transport					
Restaurant					

Air quality perceptions

25. How would you rate the indoor air quality in your home?

Good	
Average	
Poor	

25. How would you rate the outdoor air quality in your community?

Good	
Average	
Poor	

26. **Please express how much do you agree or disagree on the following statements**

	**Strongly agree**	**Agree**	**Undecided**	**Disagree**	**Strongly disagree**
Improving environment is the responsibility of every citizen					
Recycling programs should be put in place and promoted across the whole city					
I am actively involved in cleaning up the environment					
The pollution is out of my control and I cannot do anything to change it					
If I knew how to better contribute to a cleaner					

environment, I would take action					
I do not see the pollution as a health problem					

THANK YOU!
